# Repetitive negative thinking in daily life and functional connectivity among default mode, fronto-parietal, and salience networks

**DOI:** 10.1038/s41398-019-0560-0

**Published:** 2019-09-18

**Authors:** D. M. Lydon-Staley, C. Kuehner, V. Zamoscik, S. Huffziger, P. Kirsch, D. S. Bassett

**Affiliations:** 1 Department of Bioengineering, School of Engineering and Applied Sciences, University of Pennsylvania, Philadelphia, PA 19104 USA; 2 Research Group Longitudinal and Intervention Research, Department of Psychiatry and Psychotherapy, Central Institute of Mental Health, Medical Faculty Mannheim, Heidelberg University, 68159 Mannheim, Germany; 3 Department of Clinical Psychology, Central Institute of Mental Health, Medical Faculty Mannheim, Heidelberg University, 68159 Mannheim, Germany; 4 Institute Psychiatric and Psychosomatics Psychotherapy, Central Institute of Mental Health, Medical Faculty Mannheim, Heidelberg University, 68159 Mannheim, Germany; 5 Department of Electrical & Systems Engineering, School of Engineering and Applied Sciences, University of Pennsylvania, Philadelphia, PA 19104 USA; 6 Department of Neurology, Perelman School of Medicine, University of Pennsylvania, Philadelphia, PA 19104 USA; 7 Department of Physics & Astronomy, College of Arts and Sciences, University of Pennsylvania, Philadelphia, PA 19104 USA; 8 Department of Psychiatry, Perelman School of Medicine, University of Pennsylvani, Philadelphia, PA 19104 USA; 9 Santa Fe Institute, Santa Fe, NM 87501 USA

**Keywords:** Human behaviour, Depression

## Abstract

Repetitive negative thinking (RNT) is a maladaptive response to sadness and a transdiagnostic risk-factor. A critical challenge hampering attempts to promote more adaptive responses to sadness is that the between-person characteristics associated with the tendency for RNT remain uncharacterized. From the perspective of the impaired disengagement hypothesis, we examine between-person differences in blood-oxygen-level-dependent (BOLD) functional networks underlying cognitive conflict signaling, self-referential thought, and cognitive flexibility, and the association between sadness and RNT in daily life. We pair functional magnetic resonance imaging with ambulatory assessments deployed 10 times per day over 4 consecutive days measuring momentary sadness and RNT from 58 participants (40 female, mean age = 36.69 years; 29 remitted from a lifetime episode of Major Depression) in a multilevel model. We show that RNT increases following sadness for participants with higher than average between-network connectivity of the default mode network and the fronto-parietal network. We also show that RNT increases following increases in sadness for participants with lower than average between-network connectivity of the fronto-parietal network and the salience network. We also find that flexibility of the salience network’s pattern of connections with brain regions is protective against increases in RNT following sadness. Our findings highlight the importance of functional brain networks implicated in cognitive conflict signaling, self-referential thought, and cognitive flexibility for understanding maladaptive responses to sadness in daily life and provide support for the impaired disengagement hypothesis of RNT.

## Introduction

The experience of sadness is functional as it highlights a discrepancy between one’s actual state and one’s desired state^1^. Cognitive responses to sadness, however, do not always support well-being. Repetitive negative thinking (RNT) entails perseverative thinking about one’s problems or emotions and is a maladaptive response to sadness^2–4^ and a transdiagnostic risk factor for psychopathology^5–11^. The impaired disengagement hypothesis^12^ proposes that enduring negative thoughts, especially those directed towards the self, signal a cognitive conflict that leads to the disengagement of attention from negative thoughts via attentional control. From this perspective, RNT results from impaired cognitive conflict signaling and/or difficulties in enacting attentional control to divert attention away from one’s negative thoughts. In line with this perspective, deficits in inhibiting previous mental states are associated with RNT^13^ and cognitive control ability moderates the extent to which RNT follows negative moods^14^.

Three large-scale functional brain networks are increasingly incorporated into the impaired disengagement hypothesis due to their roles in conflict signaling, self-referential thought, and attentional control^12,15^. The default mode network (DMN) is characterized by a tendency to deactivate during tasks and to activate at rest, as well as during self-referential tasks^16,17^. The fronto-parietal network (FPN) is comprised of regions that have established roles in attentional control and working memory^18–20^, response selection^21^, and response suppression^22^. Due to its role in top-down, executive functions, the FPN is viewed as essential for guiding flexible, goal-directed behavior. Connectivity among the FPN and DMN provides important information about the capacity for flexible behavior, such as attentional control, with greater strength of connectivity between the DMN and the FPN associated with poorer cognitive task performance^23,24^. The salience network (SN) has two primary functions (for reviews, see refs. ^25,26^). One function relates to salience detection and the second relates to the facilitation of access to cognitive control resources (e.g., attention, working memory) following the detection of salient stimuli. The access to cognitive control is facilitated by signaling the engagement of the FPN while suppressing DMN activity^15,27^.

Alterations in DMN functioning in depression (see refs. ^28,29^ for reviews) are theorized to reflect impairments in switching between internally and externally directed thought that lead to RNT tendencies. Less work has focused on the DMN’s role in RNT in relation to both the FPN and the SN (see ref. ^30^ for review). A notable exception observed that increasing dominance of DMN activity over FPN activity is associated with increasing levels of depressive rumination^31^. Furthermore, differences emerge in the timing of activity in the insula (regions of the SN) prior to increases in FPN activity (and decreases in DMN activity) in depressed individuals relative to healthy controls, indicating a role for the SN in coordinating DMN and FPN activity in RNT (see also ref. ^32^).

We examine RNT as it occurs in response to sadness during the course of daily life in 29 remitted depressed individuals and 29 matched healthy controls. The inclusion of remitted depressed individuals ensured adequate between-person variability in sad mood and RNT, given findings that levels of RNT and negative mood are elevated in remitted depression^33^. Based on the role of the SN in switching between DMN and FPN activity upon the identification of cognitive conflict, we hypothesize that greater connectivity among the SN and the FPN, and among the SN and the DMN is associated with reduced RNT tendencies. In addition, given evidence that increased connectivity among the FPN and the DMN is associated with reduced ability to deploy cognitive functions, we hypothesize that increased connectivity among the FPN and the DMN is associated with a greater propensity to respond to sadness with RNT. We also test the moderating role of SN flexibility on the association between sadness and RNT. The SN is a uniquely flexible system, exhibiting substantial time-varying functional interactions with other functional networks^34,35^ and greater SN flexibility is associated with greater cognitive flexibility^34^. Based on the association between SN flexibility and cognitive flexibility, we hypothesize that greater SN flexibility is associated with a reduced tendency to follow sadness with RNT.

## Materials and methods

We made use of data from a study designed to provide insight into cognitive and affective function in individuals with remitted major depressive disorder. Detailed information on the larger study is available in refs. ^36–39^. The datasets analyzed and code used during the current study are available from the corresponding author upon reasonable request. The study was approved by the local ethics committee of the University of Heidelberg and conformed to the Declaration of Helsinki.

### Participants

Participants were 29 remitted depressed individuals with ≥2 episodes of major depressive disorder and 29 age-, sex-, and education-matched healthy controls without current or lifetime diagnosis for major depressive disorder. Participants were recruited by announcements in local newspapers and on the homepage of the Central Institute of Mental Health (CIMH), Mannheim, Germany. The remitted depressed individuals had to be in partial or full remission for at least two months. Exclusion criteria for all participants included clinical diagnoses of bipolar and psychotic disorders, substance dependence, current substance abuse, generalized anxiety disorder, current obsessive-compulsive, post-traumatic stress, and eating disorders according to DSM-IV as well as contraindications for magnetic resonance imaging. Psychopathology-related criteria for inclusion were assessed by a trained clinical psychologist who used the Structured Clinical Interview for DSM-IV axis I^40^. Detailed information about the sample used for the analyses is presented in Supplementary Table 1. The study was approved by the local ethics committee of the University of Heidelberg. All participants gave informed consent in writing.

### Procedure

**Fig. 1:**
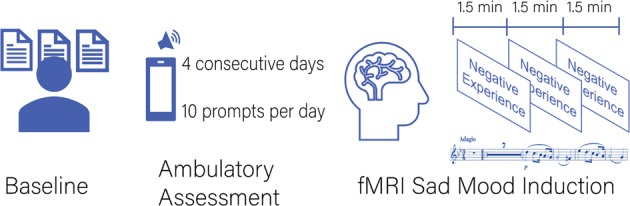
Schematic of study protocol. Participants first completed a baseline session during which demographic and clinical information was collected. Participants then underwent an ambulatory assessment protocol. The protocol was completed over four consecutive weekdays with 10 assessments per day. The beginning of each assessment was indicated by a beep, at which time the participants rated momentary mood and cognitive processes. Within 2 weeks of the baseline assessment and the ambulatory assessment, each participant underwent an fMRI session. The fMRI session included a sad mood induction. During the sad mood induction, three key words to remind participants of personal negative life events were presented for 1.5 min, each combined with sad background music (parts of Adagio in G minor by Albinoni)

We show a schematic of the procedure in Fig. 1. Demographic and clinical variables were collected at a baseline session. Affective and cognitive state variables were then measured by ambulatory assessment^41^. Ambulatory assessment was completed over four consecutive weekdays with 10 assessments per day using smartphones (HTC Touch Diamond 2) and the software MyExperienceIDE by movisens GmbH (Karlsruhe, Germany). The beginning of each assessment was indicated by a beep, at which point the participants rated momentary mood and cognitive processes. The analyses in the present study focused on reports of sadness and RNT at 10 assessments on each of the four days (a potential total of 40 assessments per person) during which participants reported on their levels of sadness and also their RNT.

The fMRI session took place within the two week period following the baseline assessment and the ambulatory assessment. Each participant underwent a sad mood induction (4.5 min) and further tasks which are not subject to the following analyses. During the sad mood induction, three key words were presented to remind participants of personal negative life events. The key words were presented for 1.5 min each during the sad mood induction scan, were chosen by participants, and were related to three negative life events produced after participants were individually assessed immediately prior to the fMRI session. Participants were also presented with sad background music (*Adagio* in G minor by Albinoni) during the sad mood induction scan.

### Measures

We made use of demographic and depressive symptom data collected during the baseline session, fMRI data collected during the sad mood induction scan, and sadness and RNT data from the ambulatory assessment.

#### Momentary sadness

Sadness was assessed during the ambulatory assessment protocol with the item “At the moment I feel sad”, rated on a scale from 0 (not at all) to 6 (very much).

#### Momentary repetitive negative thinking

RNT^42^ was assessed during the ambulatory assessment protocol with the item “At the moment, I am stuck on negative thoughts and cannot disengage from them”, rated on a scale from 0 (“not at all”) to 7 (“very much”). This measure has been used previously in ambulatory assessment studies of rumination^37,43^.

#### Depressive symptoms

At baseline, depressive symptoms during the previous 2 weeks were assessed with the self-rated Beck Depression Inventory II-Revised (BDI II^44^; German version^45^) and the Montgomery and Asberg Depression Rating Scale (MADRS^46^; German version^47^) rated by a trained clinical psychologist, both of which have shown good reliability, validity, and sensitivity to symptom changes^48^. We created a composite score for depressive symptoms by averaging the z-standardized BDI-II and MADRS scores^36,49^.

#### Blood-oxygen-level-dependent (BOLD) functional connectivity networks

A summary of our analysis of the functional imaging data is as follows: BOLD time series from the sad mood induction phase were preprocessed and an association matrix representing the functional connectivity among regions of the brain was created before connectivity indices of cognitive control were created. Additional detail is provided below.

##### Data acquisition

One-hundred and eighty T2* weighted EPI images (TR = 1.5 s, *α* = 80°, TE = 28 ms) with 24 slices (slice thickness 4 mm, voxel size 3 × 3 × 4 mm^3^, FOV 192 mm) were recorded with a 3 T TIM Trio Scanner with a 12 channel head coil (Siemens Medical Systems, Erlangen, Germany). To allow participants to adapt to the scanner environment and to reduce between-volume variance, the first 20 images of each measurement were discarded.

##### Data preprocessing

We processed the fMRI data with a preprocessing scheme based on studies that evaluated the performance of a wide variety of preprocessing pipelines in mitigating motion artifact in studies of BOLD functional connectivity^50,51^. The functional data underwent de-spiking to smooth outliers in each voxel using AFNI’s^52^ 3dDespike, rigid transformation to correct for head motion using FSL’s^53^ MCFLIRT, and slice-time correction using FSL Slicetimer to control for temporal differences in the order of the acquisition of the slices in each brain volume. Skull stripping was performed on the structural data using FSL BET. The mean functional image was computed and bias-corrected, and the skull-stripped functional image was bias-corrected. Advanced normalization tools (ANTs^54^) were used to compute the transformation parameters for the mean functional image to the high-resolution structural image. ANTs segmentation was performed to obtain a warped structural image, a skull-stripped brain mask, and masks for white matter and cerebrospinal fluid. Confound regression was then conducted. The time series was detrended by regressing the time series on the mean and the polynomial trends, up to quadratic terms. AFNI’s 3dbandpass was used to filter out very high or very low fluctuations in the signal (with a high pass of 0.01 and a low pass of 0.12). Six head motion regressors and three matter regressors (global signal, white matter, and cerebrospinal fluid), as well as their derivatives, quadratic terms, and the squares of their derivatives (36 regressors in total) were regressed from the time series. ANTs was used to warp the high-resolution structural image to the MNI template. The transformation parameters from the ANTs functional to structural co-registration and the transformation parameters from the ANTs structural to MNI co-registration were used to warp the 4D functional image to the MNI template. Finally, high variance compounds were removed^55^ using nilearn^56^.

##### Creating an association matrix

We then created an association matrix representing the strength of functional connectivity between pairs of brain regions. We defined regions of the SN, FPN, and DMN on a commonly applied parcellation scheme^57^. Coordinates of each region can be found in the supporting information. For each region, we extracted a time series of the BOLD signal separately for each individual. All regions were modeled as 10 mm diameter spheres around the center coordinates. The extracted time series were the average time series for all voxels within the sphere. We calculated the wavelet coherence matrix, *C*, using the extracted time series. Each element of *C*_*ij*_ represented the magnitude squared coherence of the scale two (0.0625–0.125 Hz) Daubechies wavelet (length 4) decomposition of the time series of region *i* and region *j*. We based our choice of frequency on previous work demonstrating sensitivity to neural processes^58–60^.

##### Between-network connectivity

We labeled regions by their putative functional systems. We calculated between-network connectivity as the mean value of the association matrix elements representing functional connectivity between the three networks of interest. This procedure resulted in three between-system connectivity indices: DMN and SN connectivity, DMN and FPN connectivity, FPN and SN connectivity.

##### SN flexibility

**Fig. 2:**
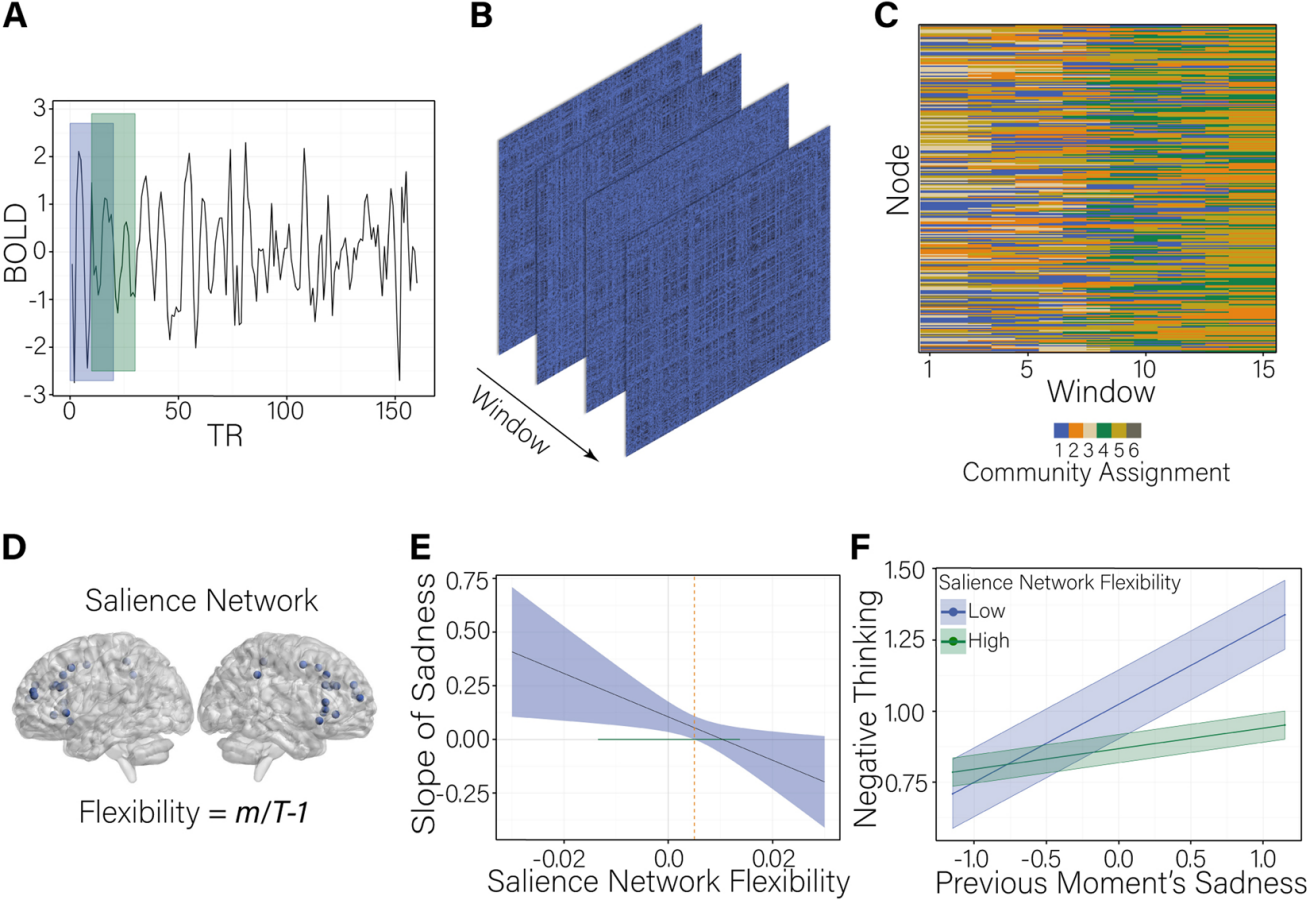
Salience network flexibility construction and results. **a** Illustrates time courses of blood-oxygen-level-dependent (BOLD) signals from one of the 264 brain regions defined by ref. ^57^. Functional connectivity between the time courses of each pair of brain regions is calculated via wavelet coherence within overlapping time windows of 20 TRs in length. **b** Illustrates connectivity matrices indicating the functional connectivity among each pair of brain regions. Four of the 15 sliding windows are illustrated. **c** Illustrates the construction of node flexibility. Multilayer modularity maximization is used to assign each brain region (node; *y*-axis) to a community (assignment indicated by color) at each sliding window (*x*-axis). Panel **d** highlights the coordinates of regions in the salience network as defined by ref. ^57^. Flexibility of an individual node captures the number of times the node changed community, normalized by the number of times the node could have changed communities. **e** Illustrates the values of salience network flexibility at which the association between previous moment’s sadness and repetitive negative thinking is significant. The dashed orange vertical line at 0.005 indicates the value of salience network flexibility at which the effect of previous moment’s sadness on negative thinking becomes non-significant. The upper bound of the region of significance for the salience network flexibility variable (0.04) is not shown as values below this bound are not observed in the sample. The range of values we observe in the sample is indicated by the horizontal green line at previous moment’s sadness = 0. The ribbon indicates the standard error of the mean. **f** Indicates the effect of previous moment’s sadness on negative thinking at low and high levels of salience network flexibility. Low and high values of between-person, sample-mean centered salience network flexibility reflect plus and minus 1 standard deviation about the mean (−0.03, 0.03). Values for the previous moment’s sadness on the *x*-axis reflect plus and minus 1 standard deviation about the mean. The slope of the simple regression of negative thinking on sadness at low levels of salience network flexibility is significant such that people with lower than average levels of salience network flexibility experienced significant increases in negative thinking following higher than usual levels of sadness at the previous measurement occasion. The slope of the simple regression at high levels of salience network flexibility is not significant. The ribbon indicates the standard error of the mean

We next created a dynamic functional connectivity index that quantifies the extent to which nodes of the SN interact with nodes outside of its community (Fig. 2a–d). The time series for each brain region was divided into *T* = 15 sliding time windows, each 20 TRs (30 s) in duration, with 50% overlap. The choice of window length was consistent with the majority of dynamic functional connectivity work to date employing 30–60 s windows, with most studies using 20 data points per window^61^. Within each window, edges between all nodes were estimated via wavelet coherence. The result was a time-ordered set of functional connectivity matrices for each subject.

We transformed the time-varying functional connectivity matrices into an ordered set of adjacency matrices, and subsequently into a multilayer network^62,63^. In this multilayer network, the graph in one time window is linked to the graph in adjacent time windows via identity edges that connect a node in one time window to the same node in neighboring time windows. We implemented multilayer modularity maximization in MATLAB^64^ and applied the procedure to each subject’s functional connectivity matrices separately. The algorithm was applied with a default structural resolution parameter of 1 and an inter-layer strength parameter of 1. As the algorithm is non-deterministic, we performed the optimization 100 times for each subject. This procedure resulted in 100 *n* × *m* matrices for each participant, where *n* is the number of nodes (264) and *m* is the number of sliding windows (i.e., 15), indicating the community allegiance of each node during each sliding window. We used these matrices to create node flexibility indices. Node flexibility captures the number of times a node changes communities across time, normalized by the number of times the node could have changed communities^58^. Using the Network Community Toolbox (http://commdetect.weebly.com), node flexibility was calculated for each subject across each pipeline as the average flexibility value across the 100 iterations of the dynamic community detection procedure. SN flexibility was calculated as the average node flexibility over all nodes within the SN.

#### Participant motion

We calculated framewise displacement (FD) of the BOLD time series to provide an index of in-scanner motion during the sad mood induction^65^. We used mean FD for each participant as a covariate in analyses. We used the percent of volumes with excessive motion to test the robustness of the findings to the exclusion of participants with excessive motion (FD > 0.05; Supplementary Tables 2 and 3).

### Data analysis

We tested the association among sadness and RNT, as well as the moderating effect of between-system functional connectivity and SN flexibility during sad mood induction, using a multilevel model to accommodate the nested nature of the data (i.e., repeated measures nested within persons)^66^. The sadness variable was lagged by one time point to create previous time point (*t-1*) sadness scores. This process allowed us to capture the temporal precedence required to increase causal inference of the association between previous moment’s sadness on current moment’s RNT. The first measurement of each day was removed to ensure that the association between the previous moment’s sadness and the current moment’s RNT was approximately equally spaced. Measurement occasions with missing data for sadness and RNT were removed. Of 2030 possible total measurement assessments (58 participants * 35 occasions accounting for lagging sadness and removing the first measurement occasion of each day), 1995 (98.28%) were available. The average time (in minutes) between assessments once the morning assessment was removed was 79.17 min (SD = 19.42). Results were robust to the inclusion of time points of ambulatory assessment data available per participant as a covariate (Supplementary Table 4) and no significant correlations emerged between completion rates and key study variables (all *p-*values > 0.05).

We then separated the sadness variable into a within-person and between-person variable to allow for both within-person and between-person inferences related to the association between sadness and RNT^67^. A time-invariant, between-person sadness variable (SadnessBW) was calculated as the arithmetic mean across each individual’s, grand-mean centered repeated measures. A time-varying, moment-level sadness variable (SadnessWN) was calculated as deviations from the between-person means. RNT was regressed on both the within-person and the between-person sadness variable, time in the study, DMN-SN, FPN-SN, DMN-FPN connectivity terms, SN flexibility, motion, sex, and depressive symptoms. Interactions among the within-person sadness variable and the functional connectivity terms were also included.

More specifically, we constructed the first level of the multilevel model as follows:

Level 1:

1 $${\mathrm{Repetitive}}\,{\mathrm{Negative}}\,{\mathrm{Thinking}}_{it} = \beta_{0i} + \beta _{1i}{\mathrm{SadnessWN}}_{i,t - 1} + \beta _{2i}{\mathrm{Time}}_{it} + e_{it}$$

where Repetitive Negative Thinking_*it*_ is RNT for person *i* at time *t*, *β*_0*i*_ indicates the average level of negative thinking for the prototypical individual in the sample, *β*_1*i*_ indicates within-person differences in RNT at time *t* associated with sadness at time *t* − 1; *β*_2*i*_ indicates the effect of time in the study on RNT; and *e*_*it*_ are time-specific residuals that were allowed to display autocorrelation (AR1).

Person-specific intercepts and associations from the Level 1 model were specified at the second level of the multilevel model, which we constructed as follows:

Level 2:

2 $$\begin{array}{l}\beta _{0i} = \gamma _{00} + \gamma _{01}{\mathrm{DefaultModeNetwork}} - {\mathrm{SalienceNetwork}}_{i}\\ +\; \gamma _{02}{\mathrm{DefaultModeNetwork}} - {\mathrm{FrontoparietalNetwork}}_{i}\\ +\; \gamma _{03}{\mathrm{FrontoparietalNetwork}} - {\mathrm{SalienceNetwork}}_{i}\\ +\; \gamma _{04}{\mathrm{SalienceNetworkFlexibility}}_{i} + \gamma _{05}{\mathrm{Motion}}_{i}\\ +\; \gamma _{06}{\mathrm{SadnessBW}}_{i} + \gamma _{07}{\mathrm{Age}}_{i} + \gamma _{08}{\mathrm{Sex}}_{i} + \gamma _{09}{\mathrm{Depression}}_{i} + u_{0i}\\ \beta _{1i} = \gamma _{10} + \gamma_{11}{\mathrm{DefaultModeNetwork}} - {\mathrm{SalienceNetwork}}_{i}\\ +\; \gamma _{12}{\mathrm{DefaultModeNetwork}} - {\mathrm{FrontoparietalNetwork}}_{i}\\ +\; \gamma _{13}{\mathrm{FrontoparietalNetwork}} - {\mathrm{SalienceNetwork}}_{i} + \gamma _{14}{\mathrm{SalienceNetworkFlexibility}}_{i} + u_{1i}\\ \beta _{2i} = \gamma _{20} + u_{2i}\end{array}$$

where the *γ* variables are sample-level parameters and the *u* variables are residual between-person differences that may be correlated with one another but are uncorrelated with the variable *e*_*it*_. Parameters *γ*_01_ to *γ*_09_ indicate the effects of person-level DMN and SAL connectivity, DMN and FPN connectivity, FPN and SAL network connectivity, SAL flexibility, motion, usual sadness, age, sex, and depressive symptoms on RNT. Parameters *γ*_11_ to *γ*_14_ indicate how between-person differences in DMN and SAL connectivity, DMN and FPN connectivity, FPN and SAL connectivity, and SAL flexibility moderated the association between the previous moment’s sadness on the current moment’s RNT.

We fit the model using lme4 in R^68^ with incomplete data being treated based on an assumption of being missing at random. We followed up on significant interactions using the Johnson-Neyman technique^69,70^ and plot simple slopes based on +1/−1 standard deviation about the mean value of the moderator variable. Statistical significance was evaluated at an *α* = 0.05. Between-network connectivity of the DMN and the SN was not a significant moderator (see Supplementary Table 5) and was removed from the final model.

## Results

Table 1 provides descriptive statistics for the variables used in the analyses. Multilevel model results are presented in Table 2.

**Table 1:** Descriptive statistics and correlations among variables used in the multilevel model

Variables	1	2	3	4	5	6	7	8	9
1. Sadness (AA)	1								
2. Negative thinking (AA)	0.84***	1							
3. Depressive symptoms	0.55***	0.61***	1						
4. DMN-FPN	−0.17	−0.21	−0.18	1					
5. DMN-SAL	−0.07	−0.18	−0.27*	0.45***	1				
6. FPN-SAL	−0.09	−0.15	−0.18	0.48***	0.51***	1			
7. SAL flexibility	−0.06	0.007	0.17	−0.27*	−0.32*	−0.43***	1		
8. Age	0.04	0.07	−0.07	0.12	0.01	0.15	−0.04	1	
9. Motion	−0.001	0.01	0.13	−0.003	−0.18	0.004	0.13	0.54***	1

Variables	1	2	3	4	5	6	7	8	9
Mean	0.00	0.91	−0.02	0.18	0.18	0.19	0.23	36.69	0.25
Standard deviation	1.07	1.02	0.95	0.01	0.01	0.01	0.01	10.66	0.11

*N* = 58

*AA* Ambulatory assessment, *Negative thinking* the person-level average of repetitive negative thinking across all measurement occasions, *Sadness* between-person version of the sadness variable used in the multilevel model, *DMN* default mode network, *FPN* fronto-parietal network, *SAL* salience network

***p < 0.001; **p < 0.01; *p < 0.05

**Table 2:** Results of the multilevel model examining associations among sadness and repetitive regative thinking, and its moderation by between-network functional connectivity and salience network flexibility

Fixed effects			
	Estimate	Standard error	*p-*value
Intercept	1.13***	0.16	<0.001
Sadness WN	0.11**	0.04	0.004
Time	−0.001	0.004	0.80
DMN-FPN	−0.77	11.04	0.94
FPN-SAL	−7.66	7.75	0.33
SAL flexibility	−7.80	13.20	0.56
Motion	−0.02	0.72	0.98
Sadness BW	0.68***	0.08	<0.001
Age	0.01	0.01	0.37
Sex	−0.29	0.15	0.05
Depressive symptoms	0.19*	0.09	0.03
DMN-FPN × Sadness WN	11.65**	3.81	0.002
FPN-SAL × Sadness WN	−11.15***	2.84	<0.001
SAL Flexibility × Sadness WN	−10.10*	4.28	0.02

Random effects			
	Estimate	Confidence interval	
Intercept	0.48	0.38–0.61	
Sadness WN	0.06	0.01–0.27	
Time	0.03	0.02–0.3	
Correlation (Intercept, Sadness WN)	0.20	−0.56–0.78	
Correlation (Intercept, Time)	0.41	0.03–0.69	
Correlation (Sadness WN, Time)	0.10	−0.43–0.57	
AR1	0.03	−0.02–0.08	
Residual	1.09	1.06–1.3	

Fit indices			
AIC	6250.58		
BIC	6373.59		

Continuous predictors were sample-mean centered and time was centered at the middle of the ambulatory assessment protocol to facilitate interpretation of the intercept. Sex was specified as a factor with 1 = male, 2 = female; the *N* = 1995 observations were nested within 58 participants

*Sadness WN* within-person deviated version of sadness, *Sadness BW* between-person version of sadness, *Depressive symptoms* composite score by averaging z-standardized BDI-II and MADRS scores, *DMN* default mode network, *FPN* fronto-parietal network, *SAL* salience network, *AR1* autocorrelation, *AIC* Akaike information criterion, *BIC* Bayesian information criterion

***p < 0.001, **p < 0.01, *p < 0.05

### FPN and SN connectivity moderates the association between sadness and RNT

**Fig. 3:**
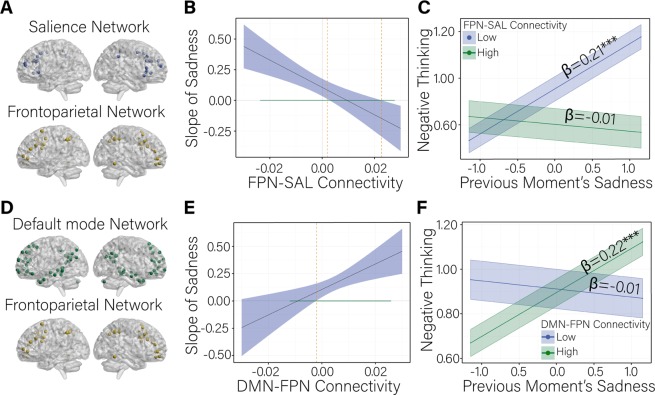
The conditional relation between sadness and repetitive negative thinking as a function of between-person differences in default mode, fronto-parietal, and salience network connectivity. **a** Highlights the coordinates of regions in the salience network and the fronto-parietal network as defined by ref. ^57^. **b** Illustrates the values of fronto-parietal network and salience network connectivity (FPN-SAL) at which the association between previous moment’s sadness and repetitive negative thinking is significant. The dashed orange vertical line at 0.003 indicates the value of FPN-SAL connectivity at which the effect of previous moment’s sadness on negative thinking becomes non-significant. The upper bound of the region of significance for the FPN-SAL variable (0.02) is also shown. The range of values we observe in the sample is indicated by the horizontal green line at slope of sadness = 0. The ribbon indicates the standard error of the mean. **c** Indicates the effect of previous moment’s sadness on negative thinking at low and high levels of FPN-SAL connectivity. Low and high values of between-person, sample-mean centered FPN-SAL connectivity reflect plus and minus 1 standard deviation about the mean (−0.01, 0.01). Values for the previous moment’s sadness on the *x*-axis reflect plus and minus 1 standard deviation about the mean. The slope of the simple regression of negative thinking on sadness at low levels of FPN-SAL connectivity is significant such that people with lower than average levels of FPN-SAL connectivity experienced significant increases in negative thinking following higher than usual levels of sadness. The slope of the simple regression at high levels of FPN-SAL connectivity is not significant. The ribbon indicates the standard error of the mean. **d** Highlights the coordinates of regions in the default mode network and the fronto-parietal network as defined by ref. ^57^. **e** Illustrates the values of default mode network and fronto-parietal network connectivity (DMN-FPN) at which the association between previous moment’s sadness and negative thinking is significant. The dashed orange vertical line at −0.002 indicates the lower bound of the region of significance on the DMN-FPN connectivity variable at which point the effect of previous moment’s sadness on negative thinking becomes significant. The upper bound is beyond the range of observed data and is not shown. The range of values observed in the sample is indicated by the horizontal green line at sadness = 0. The ribbon indicates the standard error of the mean. **f** Indicates the association between previous moment’s sadness and negative thinking at low and high levels of DMN-FPN connectivity. Low and high values of between-person, sample-mean centered DMN-FPN connectivity are defined as plus and minus 1 standard deviation about the mean (−0.01, 0.01). Values for the previous moment’s sadness on the *x*-axis reflect plus and minus 1 standard deviation about the mean. The slope of the simple regression at high levels of DMN-FPN connectivity is significant such that people with higher than average levels of DMN-FPN connectivity experienced significant increases in negative thinking following higher than usual levels of sadness at the previous measurement occasion. The slope of the simple regression of negative thinking on sadness is not significant at low levels of DMN-FPN connectivity. The ribbon indicates the standard error of the mean

We observe an interaction between FPN and SN connectivity and the association between sadness and RNT (*γ* = −11.15, *p* < 0.001). The regression of RNT on sadness is significant and positive for participants with relatively weak connectivity among these systems (Fig. 3b; *β* = 0.21 (0.05), *p* < 0.001). Participants with low levels of FPN and SN connectivity experience increases in RNT following moments of higher than usual sadness (Fig. 3c). In contrast, participants with higher levels of FPN and SN connectivity show no moment-to-moment association between RNT and sadness (*β* = 0.01 (0.05), *p* = 0.89).

### DMN and FPN connectivity moderates the association between sadness and RNT

We observe an interaction between DMN and FPN connectivity and the association between sadness and RNT (*γ* = 11.65, *p* = 0.002). The regression of RNT on sadness is positive for participants with relatively high levels of connectivity between these networks (Fig. 3e; *β* = 0.22 (0.02), *p* < 0.001). Participants with high levels of DMN and FPN connectivity experience increases in RNT following moments of higher than usual sadness. In contrast, participants with low levels of DMN and FPN connectivity show no changes in RNT following moments of higher than usual sadness (Fig. 3f; *β* = 0.01 (0.05), *p* = 0.83).

### SN flexibility moderates the association between sadness and RNT

We observe an interaction between SN flexibility and the association between sadness and RNT (*γ* = −10.10, *p* = 0.02). The regression of RNT on previous moment’s sadness is positive for participants with low values of SN flexibility (Fig. 2e; *β* = 0.21 (0.07), *p* = 0.004). Participants with low levels of SN flexibility experience increases in RNT following moments of higher than usual sadness (Fig. 2f). In contrast, participants with higher levels of SN flexibility show no moment-to-moment association between RNT and sadness (*β* = 0.04 (0.03), *p* = 0.90).

### Additional analyses

Additional analyses confirm that results are robust to (i) the removal of non-significant covariates (Supplementary Table 6), (ii) the inclusion of group status (remitted depressed versus healthy controls) rather than the continuous measure of depressive symptoms (Supplementary Table 7), (iii) the inclusion of previous moment’s RNT to account for any covariation between RNT and sadness at time *t*-1 (i.e., establishing causality in the sense of Granger; Supplementary Table 8), and (iv) including interactions with depression group indicating that results hold irrespective of group status, although we note that the sample may be underpowered to detect significant three-way interactions (Supplementary Table 9). In all cases, the moment-to-moment association between sadness and RNT is moderated by connectivity among the DMN and FPN, connectivity among SAL and FPN, and SAL flexibility in a manner consistent with the main analyses.

## Discussion

RNT is a maladaptive response to sadness. Characterizing between-person differences associated with RNT tendencies will facilitate attempts to promote more adaptive responses to sadness. Building on the impaired disengagement hypothesis^12^ and empirical support for the role of cognitive flexibility in moderating the tendency to engage in RNT^14^, we tested the moderating role of functional connectivity among brain networks associated with cognitive control^15^. We show that functional connectivity among the DMN, FPN, and SN during sad mood moderates the moment-to-moment association between sadness and RNT in daily life. Increased between-network connectivity of the DMN and the FPN, and decreased between-network connectivity of the SN and the FPN, are associated with increases in RNT following increases in sadness. We also observe that participants with flexible engagement of the SN show no tendency to increase RNT following increases in sadness. Between-network connectivity of the SN and DMN does not moderate the association between sadness and RNT. The findings are consistent with the impaired disengagement hypothesis and highlight potential roles for cognitive conflict signaling and attentional control in inhibiting RNT^13,14^. Individuals with greater ability to identify cognitive conflict and to engage executive functions may be better able to inhibit perseverative thinking and to divert attention away from their negative mood to a greater extent than individuals with impairments in these functions.

The pattern of findings for DMN and FPN connectivity, and for FPN and SN connectivity, is in line with previous work indicating that greater strength of connectivity between the DMN and FPN is associated with poorer cognitive control performance^23^ and that the SN plays a causal role in switching between FPN and DMN activity^27^. The specificity of the association for FPN and SN network connectivity and not DMN and SN connectivity, however, indicates more complex interactions among the three systems than is currently appreciated in the broader literature. Recent work provides insight into these complex interactions^71^. During an *n-*back working memory task, increased connectivity between the FPN and DMN was associated with poorer performance. Coupling both levels of activation and connectivity within a dynamical model designed to probe the association between system activity and connectivity, there was no evidence that activity of the DMN was associated with the functional coupling between the DMN and FPN. In contrast, modulating FPN amplitude impacted the strength of connectivity between DMN and FPN. These findings suggest that connectivity between the SAL and FPN may be particularly important for cognitive control, with changes in activity in the FPN in response to signals from the SAL leading to changes in connectivity between the FPN and DMN and, in turn, changes in cognitive control abilities.

A notable finding is that SN flexibility was protective against experiencing increases in RNT following increases in sadness. This finding provides further support for a putative role of cognitive flexibility in understanding maladaptive responses to depressed mood. SN flexibility captures the extent to which nodes of the SN interact with nodes in other communities. Nodes that change communities many times may moderate multiple processes and may be essential for dynamic and adaptive processes^58^. Indeed, flexibility of the SN is associated with cognitive flexibility^34^. We find that SN inflexibility is associated with increases in RNT following sadness above and beyond time-invariant (i.e., static) functional connectivity indices, in line with the increasing recognition of the time-varying nature of brain network organization^72^ and its importance for understanding human mood and cognition^59,73–77^.

### Limitations

It is important to consider the findings in light of the study’s strengths and limitations. Our measure of RNT captures perceived uncontrollability over negative thoughts. Worry and rumination are two forms of RNT that have received extensive treatment in the literature, with worry being more future-focused and rumination more past-focused^78^. Examining the content of RNT will allow insight into the specificity of our findings to worry and rumination, though it has been argued that common processes exist across different forms of RNT^79,80^. Connectivity among the DMN, FPN, and SN provides a general index of cognitive flexibility but specific cognitive functions were not examined. Working memory has emerged as a particularly important function for understanding RNT and an examination of its neural correlates in relation to RNT during the course of daily life will be an important avenue for future research. The extent to which patterns of functional connectivity during sad mood induction specifically drives the results will require further investigation, especially since emotional states modulate cognitive flexibility^81^, including in the context of remitted depression^38^.

## Conclusions

Functional connectivity among the DMN, the FPN, and the SN, as well as SN flexibility, during sad mood induction moderates the lagged, moment-to-moment association between sadness and RNT in daily life. The findings highlight the utility of large-scale functional brain networks with roles in cognitive conflict signaling, self-referential thought, and cognitive flexibility above and beyond self-report and interviewer-rated depressive symptoms in understanding the cognitive-affective sequelae of sadness. Results also indicate the feasibility and importance of considering moment-to-moment interactions between affect and cognition in daily life and encourages their consideration in cognitive control training interventions^82–84^ as well as in mindfulness-based interventions^85^ designed to reduce RNT.

## Supplementary information

Supplemental Tables

MNI Coordinates of Regions of Interest

## Data Availability

Code used throughout for data processing and analysis are available from the corresponding author.
