# Supplementary material for: Repetitive negative thinking in daily life and functional connectivity among default mode, fronto-parietal, and salience networks
Source: Transl Psychiatry. 2019 Sep 18;9:234. doi: 10.1038/s41398-019-0560-0 (PMC6751201; doi:10.1038/s41398-019-0560-0)
Supplement: Supplementary file 1 — Supplemental Tables [file 41398_2019_560_MOESM1_ESM.docx]

**Supplemental Material**

**Table S1. Demographic and Clinical Characteristics of the Sample**

|  | Mean (SD)/% | Range (min – max) |
| --- | --- | --- |
| Demographic |  |  |
| Age (Years) | 36.69 (10.66) | 19 - 55 |
| Gender (% female) | 68.97% |  |
| Education (% > 10 years) | 65.52% |  |
| Work situation (% In regular job or education) | 82.76% |  |
| Marital status (% married or living together) | 51.72% |  |
|  |  |  |
| Clinical |  |  |
| BDI-II | 7.85 (8.99) | 0 – 32 |
| MADRS | 3.72 (4.23) | 0 – 16 |
| Current psychotherapy | 8.62% |  |
| Current psychotropic medication | 12.07% |  |
| Number of previous episodes* | 3.62 | 2 - 7 |
| Duration of previous hospitalizations (weeks)^+^ | 17.31 | 2 - 75 |
| Depressive symptoms (composite score BDI-II, MADRS) | -0.02 (0.95) | -0.92 – 2.60 |

*Notes*: *N*=58; **N*=29; ^+^*N*=13; BDI-II = Beck Depression Inventory II; MADRS = Montgomery and Asberg Depression Rating Scale.

**Table S2. Multilevel Model Results with Participants with Greater than 20% of Volumes with Excessive Head Motion Removed**

| Fixed Effects | | | |
| --- | --- | --- | --- |
|  | *Estimate* | *Standard Error* | *p-value* |
| Intercept | 1.19*** | 0.18 | <0.001 |
| Sadness WN | 0.11** | 0.04 | 0.004 |
| Time | 0.00002 | 0.004 | 0.99 |
| DMNFPN | -2.19 | 11.65 | 0.85 |
| FPNSAL | -8.71 | 7.91 | 0.28 |
| SAL Flexibility | -7.88 | 13.40 | 0.56 |
| Motion | 1.28 | 1.14 | 0.27 |
| Sadness BW | 0.71*** | 0.08 | <0.001 |
| Age | 0.04 | 0.01 | 0.62 |
| Sex | -0.31 | 0.17 | 0.07 |
| Depressive symptoms | 0.14 | 0.10 | 0.15 |
| DMN-FPN x Sadness WN | 10.31* | 4.19 | 0.01 |
| FPN-SAL x Sadness WN | -10.75*** | 2.97 | <0.001 |
| SAL Flexibility x Sadness WN | -9.89* | 4.50 | 0.02 |
| Fit Indices | | | |
| AIC | 5522.07 | | |
| BIC | 5642.22 | | |

*Notes*: ****p*<0.001, ***p*<.01, **p*<.05. Sadness WN = within-person deviated version of sadness; Sadness BW = between-person version of sadness; DMN = default mode network; FPN = fronto-parietal network; SAL = salience network. Continuous predictors are sample-mean centered and time is centered at the middle of the ambulatory assessment protocol to facilitate interpretation of the intercept. Sex is specified as factor with 1=male, 2=female; *N* = 1753 observations nested within 51 participants.

**Table S3. Multilevel Model Results with Participants with Greater than 10% of Volumes with Excessive Head Motion Removed**

| Fixed Effects | | | |
| --- | --- | --- | --- |
|  | *Estimate* | *Standard Error* | *p-value* |
| Intercept | 1.25*** | 0.20 | <0.001 |
| Sadness WN | 0.10* | 0.04 | 0.02 |
| Time | 0.0003 | 0.005 | 0.96 |
| DMNFPN | -1.06 | 12.88 | 0.93 |
| FPNSAL | -8.43 | 8.81 | 0.34 |
| SAL Flexibility | -6.92 | 14.03 | 0.62 |
| Motion | 2.19 | 1.34 | 0.11 |
| Sadness BW | 0.70*** | 0.08 | <0.001 |
| Age | 0.005 | 0.09 | 0.60 |
| Sex | -0.33 | 0.18 | 0.07 |
| Depressive symptoms | 0.13 | 0.10 | 0.21 |
| DMN-FPN x Sadness WN | 11.65* | 4.95 | 0.02 |
| FPN-SAL x Sadness WN | -11.80*** | 3.52 | <0.001 |
| SAL Flexibility x Sadness WN | -10.35* | 4.90 | 0.03 |
| Fit Indices | | | |
| AIC | 5032.82 | | |
| BIC | 5151.12 | | |

*Notes*: ****p*<0.001, ***p*<.01, **p*<.05. Sadness WN = within-person deviated version of sadness; Sadness BW = between-person version of sadness; DMN = default mode network; FPN = fronto-parietal network; SAL = salience network. Continuous predictors are sample-mean centered and time is centered at the middle of the ambulatory assessment protocol to facilitate interpretation of the intercept. Sex is specified as factor with 1=male, 2=female; *N* = 1613 observations nested within 47 participants.

**Table S4. Supplemental Analysis Including Number of Ambulatory Assessment Time Points Available Per Participant as a Covariate**

| Fixed Effects | | | |
| --- | --- | --- | --- |
|  | *Estimate* | *Standard Error* | *p-value* |
| Intercept | 1.15*** | 0.16 | <0.001 |
| Sadness WN | 0.11** | 0.04 | 0.004 |
| Time | -0.001 | 0.004 | 0.80 |
| DMN-FPN | -0.51 | 10.61 | 0.96 |
| FPN-SAL | -10.54 | 7.58 | 0.17 |
| SAL Flexibility | -16.28 | 13.21 | 0.22 |
| Motion | -0.01 | 0.69 | 0.99 |
| Sadness BW | 0.64*** | 0.08 | <0.001 |
| Age | 0.01 | 0.01 | 0.26 |
| Sex | -0.26 | 0.14 | 0.07 |
| Depressive symptoms | 0.23* | 0.09 | 0.01 |
| Ambulatory Assessment Days | -0.08* | 0.04 | 0.03 |
| DMN-FPN x Sadness WN | 11.64** | 3.87 | 0.003 |
| FPN-SAL x Sadness WN | -11.22*** | 2.88 | 0.0001 |
| SAL Flexibility x Sadness WN | -10.33* | 4.34 | 0.02 |
| Fit Indices | | | |
| AIC | 6252.55 | | |
| BIC | 6381.14 | | |

*Notes*: ****p*<.001, ***p*<.01, **p*<.05. Sadness WN = within-person deviated version of sadness; Sadness BW = between-person version of sadness; DMN = default mode network; FPN = fronto-parietal network; SAL = salience network. Continuous predictors are sample-mean centered and time is centered at the middle of the ambulatory assessment protocol to facilitate interpretation of the intercept. Sex is specified as a factor with 1=male, 2=female; *n=* 1995 observations nested within 58 participants.

**Table S5. Multilevel Model Results with the Inclusion of Default Mode Network and Salience Network Connectivity**

| Fixed Effects | | | |
| --- | --- | --- | --- |
|  | *Estimate* | *Standard Error* | *p-value* |
| Intercept | 1.12*** | 0.19 | <0.001 |
| Sadness WN | 0.09* | 0.04 | 0.03 |
| Time | -0.001 | 0.004 | 0.79 |
| DMN-SAL | -0.80 | 9.48 | 0.93 |
| DMN-FPN | -0.77 | 11.76 | 0.95 |
| FPN-SAL | -7.29 | 8.26 | 0.38 |
| SAL Flexibility | -7.87 | 13.36 | 0.56 |
| Motion | -0.03 | 0.74 | 0.97 |
| Sadness BW | 0.68*** | 0.08 | <0.001 |
| Age | 0.01 | 0.01 | 0.38 |
| Sex | -0.29 | 0.15 | 0.06 |
| Depressive symptoms | 0.20* | 0.09 | 0.04 |
| DMN-SAL x Sadness WN | -2.19 | 3.33 | 0.51 |
| DMN-FPN x Sadness WN | 12.09** | 4.04 | 0.003 |
| FPN-SAL x Sadness WN | -10.22** | 3.16 | 0.001 |
| SAL Flexibility x Sadness WN | -10.60* | 4.50 | 0.02 |
| Fit Indices | | | |
| AIC | 6243.66 | | |
| BIC | 6377.83 | | |

*Notes*: ****p*<0.001, ***p*<.01, **p*<.05. Sadness WN = within-person deviated version of sadness; Sadness BW = between-person version of sadness; DMN = default mode network; FPN = fronto-parietal network; SAL = salience network. Continuous predictors are sample-mean centered and time is centered at the middle of the ambulatory assessment protocol to facilitate interpretation of the intercept. Sex is specified as factor with 1=male, 2=female; Depression Group is specified as factor with 1=healthy controls, 2=remitted depressed; *N* = 1995 observations nested within 58 participants.

**Table S6. Multilevel Model Results with Non-Significant Covariates Removed**

| Fixed Effects | | | |
| --- | --- | --- | --- |
|  | *Estimate* | *Standard Error* | *p-value* |
| Intercept | 0.88*** | 0.11 | <0.001 |
| Sadness WN | 0.11* | 0.04 | 0.004 |
| Time | -0.001 | 0.004 | 0.79 |
| DMNFPN | -4.46 | 11.15 | 0.69 |
| FPNSAL | -8.42 | 7.86 | 0.29 |
| SAL Flexibility | -4.02 | 13.36 | 0.76 |
| Sadness BW | 0.71*** | 0.08 | <0.001 |
| Depressive symptoms | 0.18* | 0.09 | 0.05 |
| DMN-FPN x Sadness WN | 11.73* | 3.81 | 0.002 |
| FPN-SAL x Sadness WN | -11.26*** | 2.84 | <0.001 |
| SAL Flexibility x Sadness WN | -10.26* | 4.28 | 0.02 |
| Fit Indices | | | |
| AIC | 6241.28 | | |
| BIC | 6347.53 | | |

*Notes*: ****p*<0.001, **p*<.05. Sadness WN = within-person deviated version of sadness; Sadness BW = between-person version of sadness; DMN = default mode network; FPN = fronto-parietal network; SAL = salience network. Continuous predictors are sample-mean centered and time is centered at the middle of the ambulatory assessment protocol to facilitate interpretation of the intercept. *N* = 2031 observations nested within 58 participants.

**Table S7. Multilevel Model Results with the Inclusion of Group Status (Remitted Depressed Versus Healthy Controls) Rather than the Continuous Measure of Depressive Symptoms**

| Fixed Effects | | | |
| --- | --- | --- | --- |
|  | *Estimate* | *Standard Error* | *p-value* |
| Intercept | 0.96*** | 0.17 | <0.001 |
| Sadness WN | 0.11** | 0.04 | 0.003 |
| Time | -0.001 | 0.004 | 0.81 |
| DMNFPN | -7.20 | 11.21 | 0.52 |
| FPNSAL | -7.89 | 7.65 | 0.31 |
| SAL Flexibility | -5.86 | 12.90 | 0.65 |
| Motion | 0.38 | 0.69 | 0.58 |
| Sadness BW | 0.67*** | 0.07 | <0.001 |
| Age | 0.004 | 0.01 | 0.57 |
| Sex | -0.34* | 0.15 | 0.02 |
| Depression group | 0.40* | 0.15 | 0.01 |
| DMN-FPN x Sadness WN | 11.91** | 3.78 | 0.002 |
| FPN-SAL x Sadness WN | -11.14*** | 2.82 | <0.001 |
| SAL Flexibility x Sadness WN | -9.93* | 4.25 | 0.02 |
| Fit Indices | | | |
| AIC | 6247.43 | | |
| BIC | 6370.44 | | |

*Notes*: ****p*<0.001, ***p*<.01, **p*<.05. Sadness WN = within-person deviated version of sadness; Sadness BW = between-person version of sadness; DMN = default mode network; FPN = fronto-parietal network; SAL = salience network. Continuous predictors are sample-mean centered and time is centered at the middle of the ambulatory assessment protocol to facilitate interpretation of the intercept. Sex is specified as factor with 1=male, 2=female; Depression Group is specified as factor with 1=healthy controls, 2=remitted depressed; *N* = 1995 observations nested within 58 participants.

**Table S8. Supplemental Analysis Including Lagged Repetitive Negative Thinking as a Covariate**

| Fixed Effects | | | |
| --- | --- | --- | --- |
|  | *Estimate* | *Standard Error* | *p-value* |
| Intercept | 1.07*** | 0.16 | <0.001 |
| Sadness WN | 0.10* | 0.04 | 0.01 |
| Repetitive Negative Thinking WN | 0.06* | 0.03 | 0.03 |
| Time | -0.002 | 0.004 | 0.69 |
| DMN-FPN | 1.97 | 10.70 | 0.85 |
| FPN-SAL | -7.58 | 7.53 | 0.32 |
| SAL Flexibility | -2.65 | 12.74 | 0.84 |
| Motion | 0.03 | 0.69 | 0.97 |
| Sadness BW | 0.71*** | 0.07 | <0.001 |
| Age | 0.01 | 0.01 | 0.29 |
| Sex | -0.27 | 0.14 | 0.07 |
| Depressive symptoms | 0.15 | 0.08 | 0.08 |
| DMN-FPN x Sadness WN | 11.37** | 3.74 | 0.002 |
| FPN-SAL x Sadness WN | -11.11*** | 2.77 | <0.001 |
| SAL Flexibility x Sadness WN | -9.92* | 4.15 | 0.02 |
| Fit Indices | | | |
| AIC | 6259.91 | | |
| BIC | 6410.86 | | |

*Notes*: ****p*<.001, ***p*<.01, **p*<.05. Sadness WN = within-person deviated version of sadness; Repetitive Negative Thinking WN = within-person deviated version of repetitive negative thinking; Sadness BW = between-person version of sadness; DMN = default mode network; FPN = fronto-parietal network; SAL = salience network. Continuous predictors are sample-mean centered and time is centered at the middle of the ambulatory assessment protocol to facilitate interpretation of the intercept. Sex is specified as a factor with 1=male, 2=female; *n=* 1995 observations nested within 58 participants.

**Table S9. Supplemental Analysis Including 3-Way Interactions Among Depression Group Status, Functional Connectivity, and the Association Among Sadness and Repetitive Negative Thinking**

| Fixed Effects | | | |
| --- | --- | --- | --- |
|  | *Estimate* | *Standard Error* | *p-value* |
| Intercept | 0.91*** | 0.23 | <0.001 |
| Sadness WN | 0.14 | 0.10 | 0.18 |
| Time | -0.001 | 0.004 | 0.81 |
| DMN-FPN | -17.36 | 18.23 | 0.35 |
| FPN-SAL | -2.77 | 10.73 | 0.80 |
| SAL Flexibility | 9.65 | 26.63 | 0.72 |
| Motion | 0.40 | 0.72 | 0.58 |
| Sadness BW | 0.65*** | 0.08 | <0.001 |
| Age | 0.005 | 0.01 | 0.55 |
| Sex | -0.36* | 0.15 | 0.02 |
| Depression Group | 0.46 | 0.25 | 0.07 |
| DMN-FPN x Sadness WN | 1.10 | 9.15 | 0.90 |
| FPN-SAL x Sadness WN | -9.26 | 4.85 | 0.06 |
| SAL Flexibility x Sadness WN | -13.81 | 12.74 | 0.28 |
| Depression Group x Sadness WN | -0.04 | 0.11 | 0.69 |
| DMN-FPN x Depression Group | 18.81 | 23.29 | 0.42 |
| FPN-SAL x Depression Group | -11.77 | 17.00 | 0.49 |
| SAL Flexibility x Depression Group | -23.23 | 30.65 | 0.45 |
| SadnessWN x DMN-FPN x Depression Group | 14.23 | 10.41 | 0.17 |
| SadnessWN x FPN-SAL x Depression Group | -4.00 | 6.45 | 0.54 |
| SadnessWN x SAL Flexibility x Depression Group | 3.00 | 13.67 | 0.83 |
| Fit Indices | | | |
| AIC | 6219.74 | | |
| BIC | 6381.79 | | |

*Notes*: ****p*<0.001, ***p*<.01, **p*<.05. Sadness WN = within-person deviated version of sadness; Sadness BW = between-person version of sadness; DMN = default mode network; FPN = fronto-parietal network; SAL = salience network. Continuous predictors are sample-mean centered and time is centered at the middle of the ambulatory assessment protocol to facilitate interpretation of the intercept. Sex is specified as factor with 1=male, 2=female; Depression Group is specified as factor with 1=healthy controls, 2=remitted depressed; *N* = 1995 observations nested within 58 participants.
